# Personalised 3D Printed Fast-Dissolving Tablets for Managing Hypertensive Crisis: In-Vitro/In-Vivo Studies

**DOI:** 10.3390/polym12123057

**Published:** 2020-12-20

**Authors:** Amjad Hussain, Faisal Mahmood, Muhammad Sohail Arshad, Nasir Abbas, Nadia Qamar, Jahanzeb Mudassir, Samia Farhaj, Jorabar Singh Nirwan, Muhammad Usman Ghori

**Affiliations:** 1 University College of Pharmacy, University of the Punjab, Lahore 54000, Pakistan; faisalmph77@gmail.com (F.M.); nasirabbas77@gmail.com (N.A.); nadiatahirkhan14@gmail.com (N.Q.); 2 Department of Pharmacy, Bahauddin Zakariya University, Multan 60800, Pakistan; sohailarshad@bzu.edu.pk (M.S.A.); jahanzebmudassir@hotmail.com (J.M.); 3 Department of Pharmacy, School of Applied Sciences, University of Huddersfield, Huddersfield HD1 3DH, UK; samia.farhaj@hud.ac.uk (S.F.); jorabar.nirwan@hud.ac.uk (J.S.N.)

**Keywords:** hypertension, hypertensive crisis, captopril, 3D printing, hot melt extrusion, pharmacokinetics, surface metrology

## Abstract

Hypertensive crisis (HC) is an emergency health condition which requires an effective management strategy. Over the years, various researchers have developed captopril based fast-dissolving formulations to manage HC; however, primarily, the question of personalisation remains unaddressed. Moreover, commercially these formulations are available as in fixed-dose combinations or strengths, so the titration of dose according to patient’s prerequisite is challenging to achieve. The recent emergence of 3D printing technologies has given pharmaceutical scientists a way forward to develop personalised medicines keeping in view patients individual needs. The current project, therefore, is aimed at addressing the limitations as mentioned above by developing fast-dissolving captopril tablets using 3D printing approach. Captopril unloaded (F1) and loaded (F2-F4) filaments were successfully produced with an acceptable drug loading and mechanical properties. Various captopril formulations (F2–F4) were successfully printed using fused deposition modelling technique. The results revealed that the formulations (F2 and F3) containing superdisintegrant had a faster extent of dissolution and in-vivo findings were endorsing these results. The present study has successfully exhibited the utilisation of additive manufacturing approach to mend the gap of personalisation and manufacturing fast-dissolving captopril 3D printed tablets. The procedure adopted in the present study may be used for the development of fused deposition modelling (FDM) based fast-dissolving 3D printed tablets.

## 1. Introduction

Hypertension is a public-health challenge due to its high prevalence and concomitant risks, including kidney and cardiovascular complications [1,2]. It has been identified as a leading cause of mortality and the third-ranked factor for DALY (disability-adjusted life-years) worldwide [1,3,4,5]. The global burden and prevalence of hypertension are rising over time driven by changes in lifestyle, population growth and ageing. It is present among one billion of the world population and is responsible for 7.1 million deaths on an average annually [6]. The World Health Organization (WHO) defines hypertension as increase level of systolic blood pressure of 140 mm Hg or higher, or diastolic blood pressure of 90 mm Hg or higher in people who are not under drug therapy. Uncontrolled hypertension may cause serious health concerns, for example, hypertensive crisis (HC) in which the systolic blood pressure is > 180 mm Hg or the diastolic blood pressure is > 120 mm Hg [7]. The HC can be classified as hypertensive emergency or hypertensive urgency depending on the end-organ involvement, including renal, cardiac and neurologic injury [8,9]. From the estimation, it is reported that approximately 1% of the patients with hypertension develop hypertensive crisis at some point during their life span, however, men are approximately affected two times more frequently than women [10,11].

To manage HC adequately formulations with rapid onset of action are imperative yet have fewer side effects. Clinically, avoidances of sudden lowering of blood pressure is recommended as this may lead to ischaemic damage in organs which may be accustomed to higher pressures leading to stroke, myocardial infarction or blindness [12]. To evade ischaemic complications, the mean blood pressure should be reduced steadily by up to ~20% during the first hour and then 5–15% over the next 23 h [13]. The Fifth Report of the Joint National Committee recommends the use of nifedipine, captopril, clonidine and labetalol in case of hypertensive emergency [14]. Among different antihypertensive agents, the oral administration of captopril, an angiotensin-converting enzyme inhibitor, is quite common in hypertensive emergencies due to its clinical effectiveness and low toxicity [15]. It imparts its clinical effect by inhibiting the conversion of angiotensin I to angiotensin II. After oral administration of therapeutic doses (12.5–100 mg), the peak plasma levels potentially achieved in 1–2 h and the antihypertensive effect is noticeable after ~60 min [16].

Antihypertensive agents can be administered via different routes. For instance, the parenteral route is employed when an immediate reduction in blood pressure is considered necessary. However, this route carries significant risks including hypertensive reaction, high cost of treatment, risk of infections, the difficulty of drug removal or reversal and need of a specialist healthcare provider for administration and continuous clinical monitoring [17]. Conversely, fast-dissolving formulations, especially those intended for sublingual or buccal administration, have attracted significant interest from researchers [18,19,20]. These formulations rapidly disintegrate in a limited volume of saliva and mostly absorbed from the vessels of sublingual mucosa. This route produces a swift onset of action in comparison to conventional tablet and capsule formulations. The extent of drug absorbed from the sublingual mucosa by-passes the hepatic first-pass metabolism, which may lead to reduce the dosage [19]. Moreover, this route may improve compliance and medication adherence in patients suffering from dysphagia [20].

Over the years, various researchers have developed captopril based fast-dissolving formulations [21,22,23]; however, primarily, the question of personalisation remains unaddressed. Moreover, commercially these formulations are available as in fixed-dose combinations or strengths, so the titration of dose according to patient’s prerequisite is challenging to achieve. In current clinical practice personalised medicines have become a reality which can go as far as to design drugs, medicines and devices which are tailored to individual patient’s pathophysiological needs ensuring its acceptance by the body. However, the obvious challenge is their manufacturing process as fabricating smaller batches of medicines, especially designed for individual patients, can be problematic, time-consuming and costly. However, the recent emergence of 3D printing technologies has given pharmaceutical scientists a way forward to develop personalised medicines and devices [24,25,26,27,28,29]. The current project, therefore, is aimed at addressing the limitations as mentioned above by developing additively manufacturing fast-dissolving captopril tablets. Briefly, hot-melt extrusion was used to develop fast dissolving filaments which then used to print the tablet formulations using fused deposition modelling (FDM) technique. Additionally, the developed formulations were then thoroughly characterised using 3D parametric surface texture, disintegration, in-vitro dissolution and in-vivo (pharmacokinetic) studies.

## 2. Materials and Methods

### 2.1. Materials

Captopril USP grade was gifted by Valor Pharma, Islamabad Pakistan. HPC SL grade (Nisso Chemical, Tokyo, Japan), PEG-6000 (Jiangsu Maoheng Chemical Co., Shijiazhuang, China), pharmaceutical grades of sodium starch glycolate and croscarmellose sodium (Hefei Joy import and export co. ltd., Hefei. China) were gifted by CCL Pharma, Lahore. Ethanol 95% (Merck, Darmstadt, Germany) was purchased from the market. For biological studies, HPLC grade solvents i.e., acetonitrile, methanol, dichloromethane, diethylether, dihydrogen potassium phosphate, and phosphoric acid were procured from Merck Germany, whereas stabilizing agent dithiothreitol was procured from Sigma-Aldirch USA. To prepared artificial saliva all the chemicals were purchased from Fisher Scientific, UK.

### 2.2. Preparation of Filaments

Blank and drug-loaded HPC filaments were prepared using single screw hot melt extruder (Filabot EX2, Barre City, VT, USA). The HPC was ground in mortar and pestle in combination with drug and other excipients accordingly to make formulations F1 to F4. F1 (blank) consisted of HPC without the drug (plasticized with 10% w/w PEG 6000) whereas F2 has 15% w/w Captopril in addition to polymer and the plasticizer. In F3 and F4 superdisintegrants, sodium starch glycolate and croscarmellose sodium were added respectively, and the final mixture has the ratio of polymer: drug: plasticizer: superdisintegrant (65:15:10:10). The mixtures were kneaded into a uniform mass by using quantity sufficient ethanol (99%) as a wetting agent. Small pellets (~1cm) were fed to the feeding hopper, and the extrusion was carried out at a speed of 15 rpm at 120 °C through nozzles having an internal diameter of 1.75 mm. The resulting filaments were stored in plastic bags placed in a desiccator at room temperature (~25 °C) until used for further investigations.

### 2.3. Physicochemical Characterisation of Filaments

#### 2.3.1. Determination of Drug Loading

The drug content per of different filaments was determined using UV-Spectrophotometer (2550 Shimadzu, Kyoto, Japan). Each filament (0.5 g) was triturated in mortar and pestle and mixed in 50 mL of distilled water and stirred until completely dissolved. The solution was filtered, and the dissolved content was determined by taking absorbance at 220 nm (the λmax of captopril). The assay was done in triplicate (n = 3), and the average values were reported.

#### 2.3.2. Differential Scanning Calorimetry (DSC)

Differential scanning calorimetry (DSC) studies were performed on powdered captopril and drug loaded filaments using TA instrument (Model Q 2000, TA Instruments, Newcastle, DE, USA). Briefly, 5–10 mg material was transferred in aluminium pan and scanned at the heating rate of 10 °C min-1 over a temperature range of 25 to 250 °C under a continuous nitrogen purge 50 mL/min. The instrument was pre-calibrated using Zinc and indium standards.

#### 2.3.3. X-ray Powder Diffraction (XRPD)

PXRD patterns of powdered captopril and drug loaded filaments using Pan Analytical diffractometer (operating at 40 kV, 40 mA, Malvern Panalytical Ltd., Malvern, UK), using Cu-Kα radiation (λ = 1.5418 Å) with a position sensitive detector. Diffraction data were collected in the range 2θ = 5°–45° with a step size of 0.02°.

#### 2.3.4. Scanning Electron Microscopy (SEM)

The surface morphology of blank and captopril loaded filaments was examined using scanning electron microscopy (SEM), (Jeol JSM-6060CV, Jeol Inc., Peabody, MA, USA). All samples were mounted onto stubs using double-sided adhesive tape and were sputter-coated for 60 s with gold/palladium (80:20) using a Quorum SC7620 Sputter Coater (Quorum Technologies, Laughton, UK), and observed under microscope [30,31].

#### 2.3.5. Mechanical Testing

Both tensile strength and Young’s modulus represents the mechanical strength (maximum force at break) and elasticity of the material, respectively. Universal material testing machine (Lloyd Lf Plus series, Ametek, Sussex, UK) was used to determine these values. Filaments of the uniform diameter without any cracks or entrapped air bubbles were selected for the study. Each extruded filament (~5 cm long) was affixed between the two jaws of the machine and tensile strength test from Nexygen MT-materials testing software was applied as the jaws moved at the speed of 25 mm/s. The stretching force (N) and elongation (mm) where filament broke were measured, and the values of tensile strength and Young’s modulus were calculated by applying the equations 1 and 2. Each measurement was recorded in triplicate, and the average value was reported.

Tensile strength = F/A(1)

where ‘F’ is the force at which the filament breaks and ‘A’ is the cross sectional area of filament.

Young’s Modulus (E) = σ/ε(2)

where ‘σ’ is the stress applied and ‘ε’ is strain i.e., change in length of filament before it breaks.

### 2.4. Fabrication of 3D Printed Tablets

The tablet (7.0 mm diameter, 2.5 mm height) was digitally designed using CAD software and was saved into STL (stereolithographic) format. The selected filaments were loaded into Makerbot Replicator 2X (Makerbot Inc., Brooklyn, NY, USA). The fabrication of matrices was carried out at extrusion temperature of 120 °C with printer platform heated at 100 °C. Other major printer settings included; layer height 0.2 mm, with 2 shells, 90 mm/S extrusion speed, 150 mm/S travelling speed, without raft support and 100% infill density.

### 2.5. Characterisation of 3D Printed Tablets

#### 2.5.1. Geometrical, Porosity, and Morphological Assessment of 3D Printed Tablets

The diameter and thickness of the printed tablets were determined using digital calliper and the porosity of the 3D printed tablets was determined using mercury intrusion porosimetry (Auto Pore IV 9500, Micrometrics, Norcross, GA, USA). Moreover, the surface morphology of the printed tablets was assessed using Jeol JSM-6060CV, Jeol Inc. Peabody, MA, USA using the method described in Section 2.3.4.

#### 2.5.2. Determination of Tablet Hardness and Friability

A general material testing machine (Testometric M500 – 50 CT, Testometric Company Ltd., Rochdale, UK) was used for the hardness testing. The method was adopted as described by Khizer et al., 2019 [28]. Briefly, the printed tablets were placed diametrically using double sided adhesive tape and force was applied by the movement of upper punch at the rate of 5 mm/min until tablet breaks and five tablets of each group was tested and an average hardness values were reported. 10 tablets of each formulation were weighed and placed in the drum of friabilator (Western Analytical Services, Lahore, Pakistan). The drum was rotated at 25 rpm for 4 min and tablets were reweighed and weight loss was determined.

#### 2.5.3. 3D parametric Surface Texture Analysis

3D parametric surface texture analysis of printed formulations was examined using Talysurf CCI 3000 optical 3D surface profiler and the method was adopted as previously described [28]. Briefly, printed tablets placed on a clean stainless steel stage using double-sided transparent tape and 1.2 × 1.2 mm^2^ area was scanned and then 3D parametric surface texture parameters were determined using MATLAB 2017 (The Math Works, Inc. Natick, Massachusetts, USA) [28,32,33,34].

#### 2.5.4. In-Vitro Disintegration Testing

Disintegration studies were carried out on both type of printed tablets (i.e., with and without super-disintegrant). Six randomly selected tablets after weighing were placed in basket rack assembly of Erweka ZT220 disintegration testing machine (Erweka, Germany). Each tablet was covered with a disc and basket assembly immersed into solution containing artificial saliva, pH 6.8, [33] at 37 °C. Time taken by all tablets to completely leave the mesh was recorded with each experiment, was conducted in triplicate (n = 3), and average values were reported.

#### 2.5.5. In-Vitro Captopril Dissolution Investigation

The captopril release from the printed tablets (F2-F4) were performed in USP dissolution apparatus II (DAWN Scientific, Pakistan) using artificial saliva, pH 6.8, (volume: 250 mL, temperature: 37 ± 0.5 °C) at a rotation speed of 50 rpm. Aliquots of 5 mL were withdrawn at time intervals of 15, 30, 45, 60 and 120 min and replaced with fresh dissolution medium in order to maintain the sink conditions. The captopril was quantified using UV Visible spectrophotometer (UV 1800 Shimadzu, Japan) at 220 nm and percent drug release was determined using standard calibration curve. Moreover, for comparative purposes in-vitro dissolution studies of captopril loaded filaments was also conducted by adopting the similar dissolution conditions described above.

#### 2.5.6. In-Vivo Pharmacokinetic Investigation

In the current study the in-vivo pharmacokinetic studies was carried out using healthy albino rabbits (∼2 Kg each) divided into four groups (I-IV) with each group having six rabbits (n = 6). All rabbits were housed individually in cages under environmentally controlled conditions (25 ± 2 °C; 44 ± 3% relative humidity). The study protocol was approved by the animal ethical committee, University College of Pharmacy, University of the Punjab, Lahore, Pakistan (vide letter No. D1528/UZ dated: 14-2-2020). Tablets printed from captopril loaded filaments without super-disintegrant (F2) and those with super-disintegrant (F3 and F4) were used for in-vivo studies in comparison to the commercially available brand (Capotene^®^, manufactured by GSK, Batch Number B 1096YAH). After overnight fasting under controlled temperature (25 °C), however, the animals had free-access to water. The group I animals was used for standard commercial formulation whereas group II-IV were administered equivalent oral doses of 3D printed tablets (F2, F3 and F4), respectively. Animals used in this investigation were anaesthetised by administering thiopentane (0.5 mg/mL), 7 mg/kg of the drug [35] and the formulations were carefully placed under the rabbit tongue. 1 mL of blood samples at time intervals 0, 0.30, 1, 1.5, 2, 3, 4, 6, 12, and 24 h were collected from rabbits in heparin containing BD Vacutainer^®^ tubes. These samples were centrifuged for 10 min at 5000 rpm and supernatant liquid was carefully transferred to eppendorf tubes and stored at −20 °C for further analysis.

Plasma determination of drug was determined using the previously reported HPLC method [36]. For the extraction of drug from plasma, 1.5 mL mixture of ether and dichloromethane (65:35) was added to 0.5 mL blank plasma along with the 40 µl dithiothreitol (DDT) solution. The samples were vortexed for 1 min and centrifuged at 10,000 rpm for 15 min. The upper layer was transferred to glass vials and evaporated to dryness with nitrogen stream at 37 °C. 100 µl of mobile phase consisting of 0.1 M dihydrogen phosphate buffer (75%) and acetonitrile (25%) (pH 2.8) along with phosphoric acid (10% of mobile phase) was added to the tubes and vortexed again for 1 min to reconstitute the dried residue. An injection of 20 µl was prepared and injected into the HPLC system and run for 15 min. The flow rate of mobile phase was maintained at 1ml/min with UV detector set at λmax of 205 nm. The calibration curve was constructed from spiked plasma sample for concentration of 0.10, 0.25, 0.50, 1.0, 5.0, 10.0, and 20.0 µg/mL whereas limit of detection and limit of quantification were 15 ng/mL and 50 ng/mL respectively. PKSolver program, an add-in macro for Microsoft Excel^®^, was employed for the calculation of the different pharmacokinetic parameters [37].

## 3. Results and Discussion

### 3.1. Development and Characterisation of Filaments

**Figure 1:**
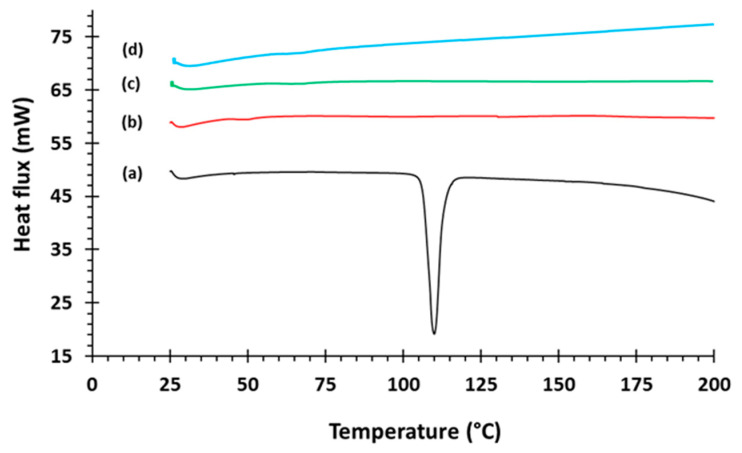
Differential scanning calorimetry (DSC) pattern of (a) captopril API (b) F2, (c) F3, and (d) F4.

Captopril unloaded (F1) and loaded (F2, F3, and F4) filaments without (F2) and with the aid of superdisintegrants (F3 and F4) were successfully extruded using HME. The captopril loading (15% w/w) in extruded filaments (F2–F4) was within the pharmacopoeial assay limit, Table 1. The DSC trace of pure captopril exhibited a sharp endothermic peak at 107 °C which is corresponding to the melting point of the drug (Figure 1). The sharpness of this peak suggests the crystalline nature of the pure drug. DSC thermograms were also acquired for captopril loaded filaments (F2–F4) to investigate any drug-polymer interactions and it is confirmed that there was no crystalline drug evident in the extruded filament (Figure 1).

**Figure 2:**
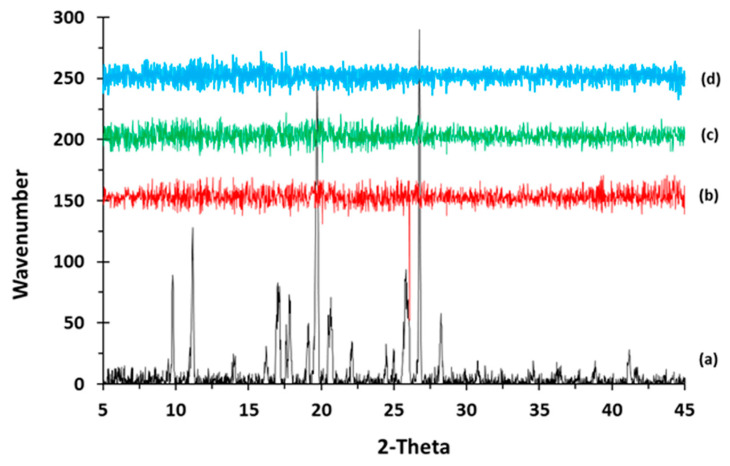
X-ray powder diffraction (XRPD) pattern of (a) captopril API (b) F2, (c) F3, and (d) F4.

**Figure 3:**
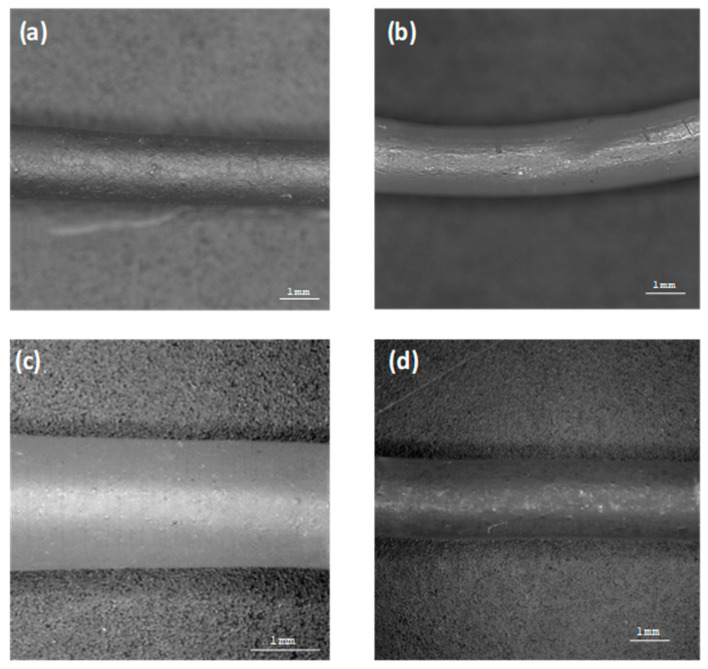
Scanning electron microscopy (SEM) pictures of extruded filaments: (**a**) F1, (**b**) F2, (**c**) F3, and (**d**) F4.

Moreover, the crystalline structure of captopril was confirmed by XRD analysis which showed multiple high-intensity peaks (Figure 2). However, when the drug loaded filaments (F2–F4) were analysed high intensity multiple peaks of captopril were not evident which is likely due to the formation of a solid dispersion that might have masked the crystalline structure of the drug (Figure 2) [38,39]. Overall, XRD results were consistent with the DSC profiles, thus, both methods showed that captopril has a crystalline structure, and whereas drug loaded filaments have amorphous nature. SEM micrographs of HPC filaments of all formulation types showed cylindrical shapes (Figure 3). The outer surfaces of these filaments were found smooth and without any visible fine powder aggregation caused by drug.

To assess the compatibility of the extruded filaments for use in the 3D printing of tablets, the mechanical properties of the extruded filaments were analysed using a three-point bend test and are presented in Table 1. The tensile strength of all the filaments ranged from 19.81 MPa to 20.16 MPa, although the standard deviations found the variations to be statistically insignificant. On the other hand, Young’s modulus and stiffness of the F1 filament were found to be substantially greater than the other three filaments and statistically significantly greater than F3 and F4 filaments. These properties reveal comparable and, in some cases, significantly greater mechanical properties to other drug-loaded polymer-based filaments described in the literature [28]. Such properties demonstrate the feasibility of the extruded filaments for successful use in 3D printing applications as filaments that are too brittle may potentially crumble, whereas filaments that are too soft may lead to printing failure due to them being squeezed aside by the feeding gear [40].

### 3.2. Development and Characterisation of 3D Printed Matrix Tablets

#### 3.2.1. Content Uniformity, Hardness, Porosity, and Friability of Printed Tablets

All the captopril loaded filaments (F2–F4) were successfully employed to develop 3D printed tablet formulations and then characterised as detailed in Table 2. It is evident from the results that the thickness, diameter and weight are robust and importantly there were no statistically significant differences. Moreover, the drug loading in the printed tablets was within the pharmacopoeial assay limit (95–105%) [41]. To understand the structural quality of the formulations, porosimetry was carried out. F2 (2.4 ± 0.5%) formulation showed the lowest porosity followed by F4 (2.1 ± 0.3%) and F3 (1.7 ± 0.4%), Table 2. Additionally, the printed tablets exhibited high degree of hardness with no signs of tablet friability, Table 2. All formulations have shown hardness (>10 Kg/cm2) which is higher than the upper limit of usual hardness tester [42]. Both higher degree of tablet hardness and friability indicate the strength of printed tablets to any mechanical breaking or fracture. Uniformity in hardness values among all formulations is attributed to the identical 3D structure and design of these tablets.

#### 3.2.2. Morphological Analysis of Printed Tablets

**Figure 4:**
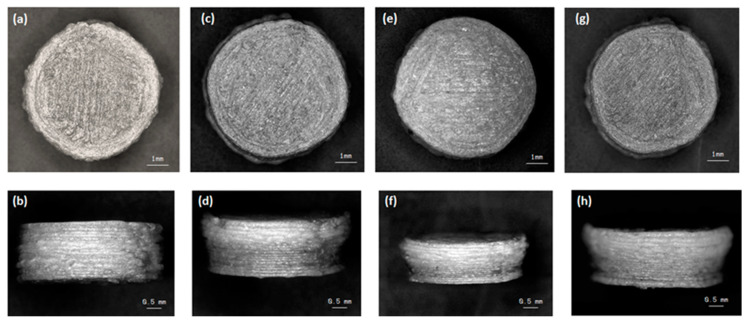
SEM micrographs of 3D printed captopril tablets with top and side view (**a**,**b**) F1, (**c**,**d**) F2, (**e**,**f**) F3, and (**g**,**h**) F4.

The SEM micrographs of the 3D printed tablets are displayed in Figure 4. These images reveal the layering pattern on the surface of the tablet due to the printing pattern of the FDM resulting in an irregular tablet surface (Figure 4a,c,e,g) and are comparable to images obtained by other researchers using the same method [29,43]. Furthermore, Figure 4b,d,f,h shows a side profile of the tablets which clearly demonstrates the layer-by-layer printing pattern of the FDM technique with layers of uneven size and shape. Moreover, the lack of a visible single particulate on the tablets indicates that the tablets were constructed in a continuous motion with homogenous compositions [44]. Nevertheless, these images confirm the successful development of tablets from the extruded filaments, albeit comprising a surface with greater irregularities than a compressed tablet.

#### 3.2.3. 3D Parametric Surface Texture Analysis of 3D Printed Tablets

**Figure 5:**
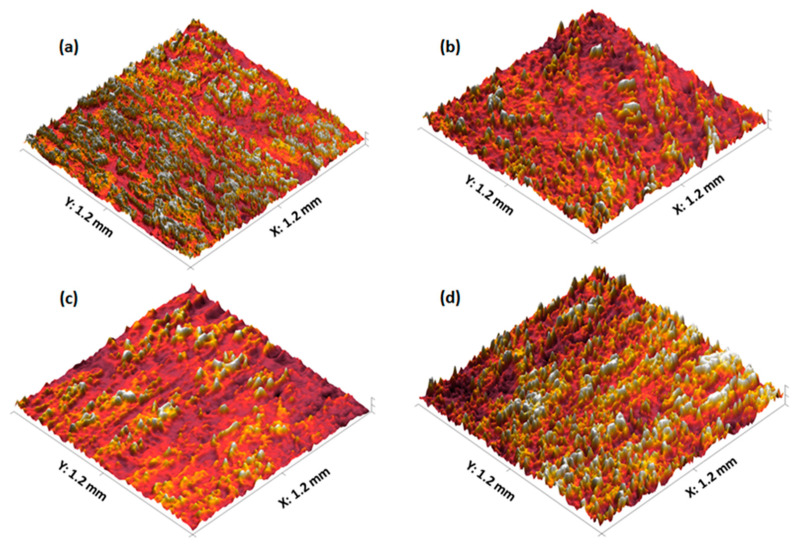
Surface texture images of 3D printed captopril tablets: (**a**) F1, (**b**) F2, (**c**) F3, and (**d**) F4.

The surface textures of the 3D printed tablets were analysed using a profilometer and the obtained images are displayed in Figure 5a–d. Upon topographical analysis, the surface of F1 comprises an even distribution of small peaks and valleys, whereas F4 displays a greater quantity of large peaks and valleys (Figure 5a,d). Moreover, Figure 5b,c reveals the presence of moderate sized peaks and valleys in localised areas on the surface of F2 and F3, respectively. These observations were also supported by quantitative surface texture analysis which included the measurement of multiple amplitude parameters (Table 3). Of these parameters, the average roughness (Sa) and the root mean square roughness (Sq) provide an overall measurement of the surface texture and reveal the greatest surface roughness on the F4 tablet and lowest surface roughness on the F1 tablet. This trend was also evident upon analysis of the maximum peak height (Sp) and maximum height of the surface (sum of height of highest peak and height of deepest valley; Sz) which were greatest for the F4 tablet followed by F3, F2, and F1. Conversely, the tablet surface with the deepest valley (Sv) was found to be on the F1 tablet. However, it is importamnt to note that these differemce were statistically insignificant. Furthermore, the kurtosis coefficient (Sku) revealed a leptokurtic distribution curve (Sku < 3) indicating the presence of inordinately high peaks and/or deep valleys and the skewness (Ssk) of the surface texture profiles indicated the predominance of valley structures on the surface of all tablets (Ssk < 0) [45].

#### 3.2.4. In-Vitro Disintegration and Dissolution Studies

**Figure 6:**
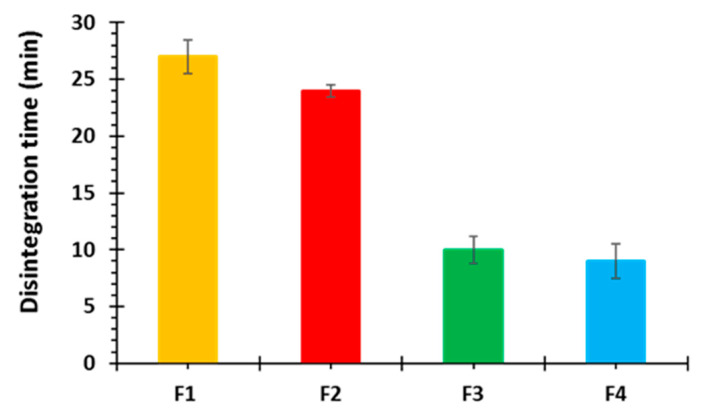
Disintegration profiles of 3D printed captopril tablets (n = 3).

Owing to it specially woven structure the printed tablets usually undergo disintegration at a slower rate compared to its corresponding filaments which present a larger surface area for disintegrating medium. The order of disintegration in 3D printed tablets was noted as F1 ˂ F2 ˂ F3 ˂ F4 based on the composition of the formulation. Blank tablets (F1) disintegrated completely within 27 min, Figure 6. It was slower because no additive was there to facilitate the penetration of disintegrating medium into the compact tablet matrix or to impart solubilising effect. Whereas drug loaded tablets containing plasticizer alone (F2) exhibited small improvement in disintegration time which was recorded as 24 min. However formulations with superdisintegrant added showed a rapid tablet disintegration i.e., 10 min with sodium starch glycolate (F3) and 9 min with croscarmellose sodium based formulation (F4), Figure 6.

**Figure 7:**
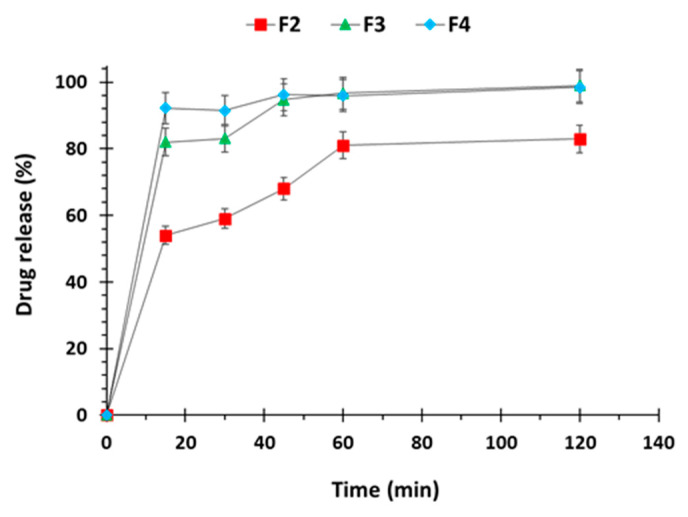
Dissolution profiles of captopril loaded filaments (n = 3).

Dissolution data from both the extruded filaments and 3D printed tablets was found to be consistent with the formulation composition. Rapid drug release was seen in filaments compared to printed tablets primarily owing to the increased surface area of these filaments. Among the filaments F2 exhibited drug release of ~55% at 15 min, whereas formulations containing superdisintegrants F3 and F4 exhibited rapid drug release of ~80 and 90%, respectively, at the same time interval. In formulations, without disintegrant drug release was complete at 1 h time interval whereas in formulations with disintegrants complete drug dissolution was achieved in 30 min as evident form Figure 7. This faster drug release is attributed to superdisintegrants i.e., sodium starch glycolate and croscarmellose sodium which absorb water and swells, leading to rapid disintegration of corresponding filaments.

**Figure 8:**
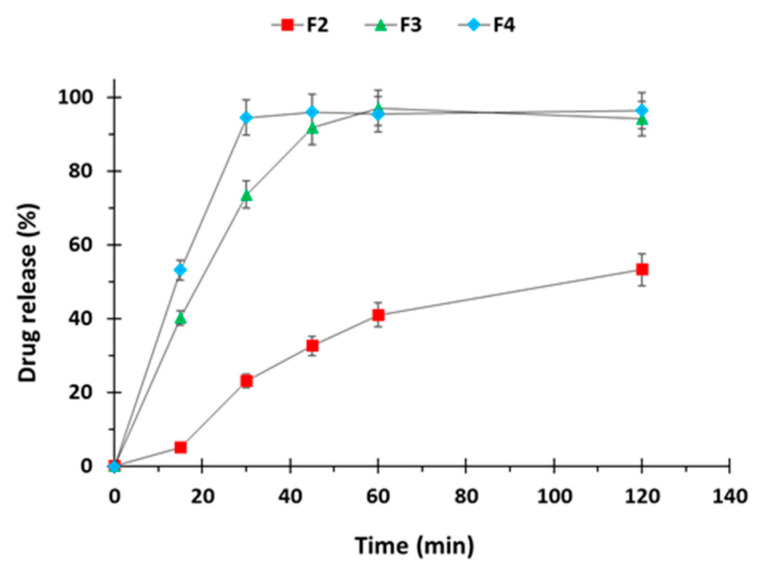
Dissolution profiles of 3D printed captopril tablets (n = 3).

Among the 3D printed tablets drug release was slower compared to corresponding filaments. In tablets containing drug only (F2) ~5 % drug was released within initial 15 min whereas in printed tablets having sodium starch glycolate (F3) drug release was higher up to 40% and even higher i.e., 50% with croscarmellose sodium based tablets (F4) for the similar time interval as seen in Figure 8. Drug release from F2 tablets was only 50% in 2 h of dissolution whereas in F3 and F4 tablets showed almost complete release in this time.

#### 3.2.5. In-Vivo Pharmacokinetic Studies

**Figure 9:**
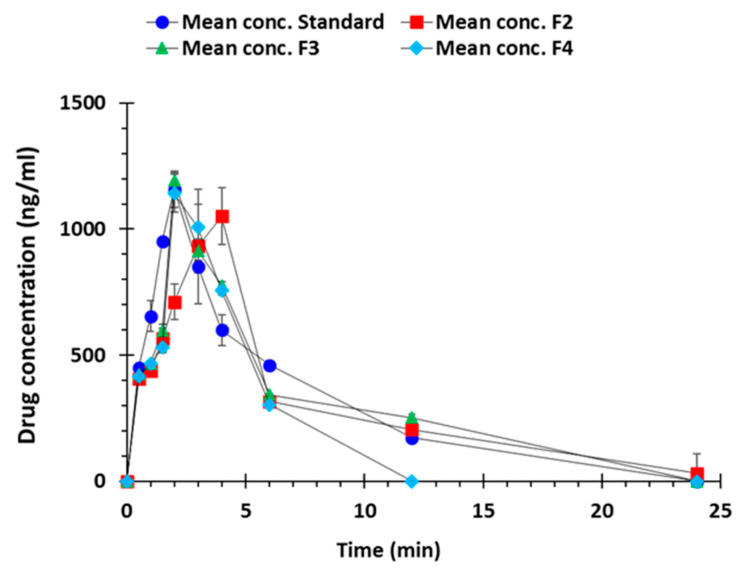
Drug absorption vs. time profiles of standard, F2, F3, and F4, 3D printed captopril tablets (n = 3).

The plasma level time curve of different formulations (F2, F3, and F4) has been compared to fast dissolving standard formulation (Capoten^®^) in Figure 9. It depicts that F3 and F4 formulations containing superdisintegrants i.e., sodium starch glycolate and croscarmellose sodium respectively has underdone faster in-vivo drug release similar to standard formulation whereas F2 formulation having no super-disintegrant constituent showed extended drug release.

The standard showed AUC values of 6005 ng/mL·h whereas among the 3D printed formulations F2 exhibited the highest mean AUC of 7039 ng/mL·h followed by F3 (5836 ng/mL·h) and F4 (4007 ng/mL·h), Table 4. These valuses were statiscally different when analysed using analysis of variance. Peak plasma concentration (Cmax) of the standard drug was 1158 ng/mL while in 3D printed formulations, F4 had the highest Cmax value of 1138 ng/mL and F2 had the lowest i.e., 1051 ng/mL. These values had statistically nonsignificant differences. Tmax increased in F2 formulation (without super-disintegrant) up to 4 h compared to standard (2 h) as it showed the extended release, however in F3 and F4 formulations (containing superdisintegrants) Tmax was again reduced to 2 h. Highest mean residence time (MRT) was noted with F2 (12 h) followed by F3 (7.66 h), Standard (6.5 h), and F4 (4.7 h). The higher MRT is attributed to the corresponding low clearance values suggesting increased absorption window. Moroever, these values showed statistically nonsignificant differences.

## 4. Conclusions

The present study has successfully exhibited the utilisation of additive manufacturing approach to mend the personalisation gap of developing fast dissolving captopril 3D printed tablets. Moreover, the present study demonstrated the inclusion of superdisintegrants to modulate the drug release profiles of the printed tablets. The presence of superdisintegrants has improved the overall in-vitro drug release and tablet disintegration profiles which is also demonstrated by the corresponding improvement in in-vivo pharmacokinetic parameters. Overall, this study confirmed the successful fabrication of personalised captopril printed formulation intended for hypertensive crisis having analogous in-vivo performance to a commercial product. Furthermore, the present study has also established the practicality of the FDM technique, providing a simple straight-forward solution to develop personalised formulations in a time and cost-effective way addressing challenges confronted by conventional manufacturing techniques.

## Figures and Tables

**Table 1:** Drug loading and mechanical properties of filaments.

Formulation	Tensile Strength (MPa)	Young’s Modulus (MPa)	Stiffness (kN/m)	Drug Loading (%)
F1	20.13 ± 0.38	1931.5 ± 193.1	134.8 ± 11.8	-
F2	19.81 ± 0.25	1727.8 ± 434.3	111.7 ± 26.6	98.65 ± 0.77
F3	19.95 ± 0.34	1468.9 ± 109.8	89.97 ± 6.72	97.43 ± 1.2
F4	20.16 ± 0.26	1475.3 ± 279.6	86.48 ± 5.2	96.50 ± 0.35

**Table 2:** Geometrical and drug loading properties of 3D printed tablets.

Formulation	Weight (mg)	Diameter (mm)	Height (mm)	Drug Loading (%)	Breaking Strength (N)	Porosity (%)	Friability (%)
F1	175.0 ± 0.75	7.00 ± 0.10	2.13 ± 0.11	-	385.1 ± 11.5	1.6 ± 0.6	0
F2	175.2 ± 0.80	7.05 ± 0.12	2.15 ± 0.15	97.15 ± 1.2	411.3 ± 19.3	2.4 ± 0.5	0
F3	175.1 ± 1.1	7.02 ± 0.15	2.20 ± 0.10	96.77 ± 0.8	406.4 ± 21.3	2.1 ± 0.3	0
F4	175.4 ± 0.5	7.10 ± 0.18	2.22 ± 0.12	98.10 ± 1.1	409.9 ± 6.98	1.7 ± 0.4	0

**Table 3:** 3D parametric surface texture analysis of 3D printed captopril tablets (n = 3).

Parameter	F1	F2	F3	F4
Sa (μm)	20.11 ± 4.25	24.19 ± 2.14	22.16 ± 1.54	25.19 ± 3.31
Sq (μm)	25.29 ± 6.84	30.15 ± 3.11	31.25 ± 2.88	33.22 ± 2.44
Sz (μm)	57.83 ± 5.92	61.95 ± 6.86	67.59 ± 5.12	75.95 ± 6.11
Sp (μm)	24.16 ± 4.21	39.26 ± 2.49	41.26 ± 4.16	44.29 ± 6.12
Sv (μm)	33.67 ± 5.22	22.69 ± 7.15	26.33 ± 7.19	31.66 ± 6.05
Sku (μm)	4.21 ± 0.95	4.55 ± 1.11	4.88 ± 0.89	5.11 ± 1.21
Ssk (μm)	−0.414 ± −0.48	−0.29 ± −0.11	−0.42 ± −0.15	−0.36 ± −0.10

**Table 4:** Pharmacokinetic parameters of 3D printed captopril tablets (n = 3).

Parameter	Standard	F2	F3	F4
T_max_ (h)	2.00 ± 0.00	3.67 ± 0.52	2.00 ± 0.00	2.17 ± 0.41
C_max_ (ng/mL)	1158.45 ± 71.67	1054.12 ± 64.36	1117.02 ± 32.46	1175.11 ± 21.65
AUC_0–24h_ (ng/mL·h)	6004.88 ± 125.49	7039.39 ± 931.58	5836.30 ± 102.51	4006.75 ± 58.66
MRT (h)	6.51 ± 0.76	11.98 ± 1.98	7.66 ± 0.28	4.70 ± 0.08
